# ​Evaluating the Effectiveness of Group Sessions with Pre-recorded Presentations on Digital and Postpartum Health Literacy Among Arab American Women

**DOI:** 10.1007/s10995-026-04238-3

**Published:** 2026-02-26

**Authors:** Israa Al-Jumaa, Ghina Sabbagh, Noor Abushaban, Denis Hulett, Heike Thiel de Bocanegra

**Affiliations:** 1 Department of Biological Sciences, University of California, Irvine, CA 92697 USA; 2 Department of Public Health, University of California, Irvine, CA 92697 USA; 3 Department of Molecular Biology and Biochemistry, University of California, Irvine, CA 92697 USA; 4 University of California, 490 Illinois St, San Francisco, CA 94158 USA; 5 Department of Obstetrics and Gynecology, UCI School of Medicine, University of California, Irvine, CA 92697 USA; 6 Department of Population Health and Disease Prevention, Joe C. Wen School of Population & Public Health, University of California, Irvine, CA 92697 USA

**Keywords:** Arab Americans, Immigrants, Refugees, Postpartum health, Reproductive health, EHealth literacy

## Abstract

**Introduction:**

Arab refugees, immigrants, and migrants (RIMs) experience barriers in accessing accurate health information. To combat this, the Refugee Reproductive Health Network (ReproNet) developed Arabic, culturally concurrent health literacy sessions that included pre-recorded presentations on postpartum care. We evaluated the impact of these group sessions on postpartum knowledge and health literacy in the Arab RIM community in Southern California.

**Methods:**

We recruited a convenience sample of 51 Arab RIM**s **through community organizations and the ReproNet client list for one in-person and three virtual informational sessions on postpartum care. We administered pre- and 2–4 week post-session surveys to measure e-Health Literacy (eHL) and Postpartum Health Literacy (PHL). We conducted descriptive analyses and t-tests to assess mean differences using SAS 9.4.

**Results:**

Of the 51 attendees, 47 participants completed both the pre- and post-tests. Most participants were college-educated, single, and had never had a live birth. The average age was 39.3 years. We observed a significant increase in both eHL and PHL after the informational sessions. Females under the age of 35 had improved more in PHL than those over 35. Nulliparous females had a lower PHL baseline than parous females.

**Discussion:**

Group health literacy training sessions that implement culturally concurrent methods, including pre-recorded presentations, are effective in increasing postpartum knowledge and health literacy in the Arab RIM community, regardless of parity. Enhancing PHL has the potential to reduce the risks of postpartum complications, such as postpartum depression (PPD), and can potentially contribute to postpartum females’ mental and physical health.

## Significance

This pilot project demonstrated the effectiveness of group sessions on postpartum care that use pre-recorded presentations prepared by bi-cultural experts. Postpartum literacy sessions and discussion guides for facilitators on postpartum care ensure quality while ensuring the comfort of clients to discuss sensitive topics in a group setting. Improved health knowledge and health literacy regarding postpartum care have the potential to improve females’ well-being in the Arab RIM community and serve as a model for other reproductive health topics and other immigrant groups.

## Introduction

The Arabic-speaking community in the United States has grown significantly in the last several years due to circumstances in Arab countries, such as Syria, Iraq, and Palestine (Harjanto & Batalova, 2022). In 2019, 52% of all U.S. immigrants were Middle Eastern and North African (MENA); 62% of them became U.S. citizens (Harjanto & Batalova, 2022). Moreover, California has the most immigrants in comparison to other states (Statista Research, 2022).

As the number of Arab refugees, immigrants, and migrants (RIMs) increases, we need more culturally appropriate material to support females’ reproductive health in Arab RIM communities. Cultural appropriateness plays a critical role in improving health education by fostering trust, ensuring relevance, and reducing barriers to understanding and engagement, particularly in communities with strong cultural norms surrounding reproductive health (Brach & Fraserirector, 2000; Singleton & Krause, 2009). Arab females strongly prefer to receive health education from a health professional (Racine & Isik Andsoy, 2022); however, the availability of Arab-speaking health professionals is limited. Hence, we aimed to test the use of pre-recorded presentations on postpartum care that were developed and recorded by Arab health professionals as part of educational sessions.

Sex education, among other reproductive health topics, is not discussed openly in the Arab community (Hussain et al., 2019). Lack of communication about reproductive health raises the concern that the Arab RIM community may not be well-informed about reproductive health topics such as postpartum care (Alhasanat et al., 2017). Among immigrant Arab females, 36% were at a higher risk of developing postpartum depression (PPD), with lack of social support and life stress as notable risk factors (Alhasanat et al., 2017). In Canada, mothers from the Middle East and North Africa (MENA) were found to be 51% more likely to develop gestational diabetes compared to Canadian-born mothers (Relative Risk = 1.51) (Côté-Corriveau et al., 2025). A U.S. study found foreign-born non-Hispanic White MENA mothers had 44% greater odds of delivering low-birth-weight infants compared to non-MENA mothers, even after adjusting for prenatal factors (OR 1.443, *p* < 0.001) (Moustafa et al., 2024).

Postpartum peer navigation programs have been shown to increase retention in postpartum care, improve contraception uptake, enhance depression screening, and reduce emergency department visits, demonstrating that culturally tailored education and support can produce better maternal outcomes in immigrant communities (Alsamman et al., 2025). Examining health disparities within the Arab or MENA population is challenging due to limited data across most federal and state data collection systems (Moustafa et al., 2024). MENA females often have reduced knowledge of PPD due to cultural stigma, language barriers, and limited access to culturally appropriate education, which may contribute to underdiagnosis and delayed treatment (Alhasanat & Fry-McComish, 2015; Alsamman et al., 2025; Moustafa et al., 2024). In addition, studies in other immigrant populations, such as Latina immigrant mothers, suggest that increasing knowledge of postpartum care can reduce anxiety and promote better health-seeking behaviors, which could lead to improved maternal and infant health outcomes (Platt et al., 2023).

One barrier to health awareness is that most refugees and immigrants come to the U.S. with limited English skills, hampering healthcare access for Arab RIMs in the initial years after resettlement in the United States (Hussain et al., 2019; Alhasanat et al., 2017; Al-Jumaili et al., 2020). The Refugee Reproductive Health Network (ReproNet), an academic-community partnership, aims to engage in dialogue with refugee females and communities to increase awareness of refugee reproductive health issues. (ReproNet, 2021). Priority groups are recent arrivals, including RIMs from Arab countries, such as Syria and Iraq. Due to the scarcity of trained female facilitators who can lead groups on sexual reproductive health topics, ReproNet has worked with Arab refugees and developed materials in order to provide vital information to females, which includes a YouTube channel and a digital library in Arabic, English, and other languages. These resources include a 60-minute Arabic talk show on female hygiene and c-sections (LightHouse Refugee Reproductive Health Segment [English & Arabic], 2022) and audio-recorded PowerPoint presentations on topics such as menopause and birth control options (ReproNet, 2021). These pre-recorded presentations can be used in individual and group health education settings to reduce the need for an interpreter, and they have shown effectiveness in increasing health literacy (Toolkits – ReproNet, 2025).

This study assesses the implementation of culturally sensitive and bilingual education sessions in the Arab RIM community in Southern California, which integrates pre-recorded presentations. We aimed to determine whether these education sessions on postpartum care can enhance the knowledge and health literacy of postpartum care among females and improve their well-being in the Arab RIM community. Our audience includes adult Arab RIM community members interested in postpartum care education, such as those planning to become pregnant, currently pregnant, a loved one of a pregnant individual, or simply seeking to improve their knowledge of the subject. We aim to reduce the gap in postpartum care education and welcome all community members to these educational sessions. The Arab RIM community may benefit most from our materials, which are culturally sensitive and allow females the option to choose whether they would like their partners to attend the sessions with them.

## Methods

The study received IRB approval #1443 from the University of California, Irvine. The study was conducted by hosting four group educational sessions on reproductive health literacy with a focus on postpartum care. These sessions included the use of Arabic pre-recorded presentations and a discussion session. Sessions were facilitated and led by a bilingual, trained Iraqi health educator and a science baccalaureate. We administered a survey before the session and a follow-up survey 2–4 weeks after the session.

To recruit Arab RIM participants, we reached out to community-based organizations (CBOs), Islamic Centers, Health Centers, ReproNet contact list, and social media pages. We distributed a flier with session information, a stipend offer, registration instructions, and the event’s time and location. The eligibility criteria included being an Arab refugee, immigrant, or migrant over 18 years of age. While the outreach targeted female participants, four male partners accompanied participants, and with the consent of the other attendees, were permitted to participate in the session. A ten-dollar gift card was offered to participants upon completion of each pre-survey and post-survey. Upon registration, participants received the pre-test via REDCap to complete prior to or at the beginning of the session (Harris et al., 2019; Harris et al., 2009).

To prepare for the sessions, a team of bilingual and bicultural ReproNet scholars translated and recorded the ReproNet presentation on postpartum care from English to Arabic. Translations and recordings underwent ReproNet review for quality and medical accuracy, using the Patient Education Assessment Tool and ReproNet content experts (PEMAT, 2020). Each session presented the Arabic pre-recorded presentation and was followed by a guided question-and-answer discussion in Arabic and English. The postpartum session addresses the content of the American College of Obstetrics and Gynecology (ACOG’s) postpartum bundle: Wound healing, breastfeeding, PPD prevention, family planning, and management of chronic diseases (Committee 2018). Participants attended the postpartum care session, either in person for two hours or virtually via Zoom for one hour. The in-person session was hosted at the Islamic Center of Irvine (ICOI). The Question and Answer component was offered in Arabic and English; however, depending on participants’ preference, we prioritized one language over the other or used both.

We followed up with participants within 2–4 weeks after the session via phone and/or email to complete the post-survey. Creating a welcoming environment and having culturally concordant researchers could have potentially contributed to participants’ willingness to share contact information and complete the follow-up survey.

The survey included questions focused on assessing demographics, e-Health Literacy (eHL) using the e-Health Literacy Scale (eHEALS), and Postpartum Health Literacy (PHL) using a scale that we developed, which included topics of maternal health and postpartum care (Norman & Skinner, 2006). Responses used a 5-item scale where 1 indicated the lowest (representing strongly disagree or not at all) and 5 indicated the highest (representing strongly agree or always). In the frequency scale, the “prefer not to respond’’ answer choice was considered as a missing value statistically.

A set of eHL questions assessed participants’ confidence levels in finding online health resources and the ability to understand, evaluate, and make health decisions using them. Maternal care and postpartum care questions evaluated PHL that included females’ health, pregnancy, contraceptives, and postpartum care. The six eHL questions are from the eHEALS, and the other ten questions are from the PHL Scale we developed.

We analyzed survey results using descriptive, bivariate analyses, and multivariate regression analysis using SAS 9.0. We calculated the Cronbach’s alpha coefficient to assess the internal reliability of the survey questions and performed one-sample t-tests to compare the pre-test and post-test responses. P-values < 0.05 were considered statistically significant.

## Results

All participants were of MENA descent; most of whom were Iraqis (40.4%) (Table 1). Most participants were under 35 years of age, college-educated, single, and had no previous live births. Participants reported their age in three predefined categories: 18–34 years (*n* = 26), 35–49 years (*n* = 5), and 50 years and older (*n* = 16). The estimated mean age of the sample is approximately 39.3 years.

**Table 1:** Participant demographics (*n* = 47)

Demographics	Number	Percentage%
Nativity		
Iraq	19	40.4
Syria	8	17.0
U.S.	7	14.9
Other Arab Countries	13	27.7
Age		
18–24	21	44.68
25–44	10	21.28
50+	16	34.04
Education		
6–12 years/High school graduates	10	21.3
Some college/College degree	37	78.7
Race/Ethnicity		
Middle Eastern/North African	41	87.2
Other (White, Asian)	6	12.8
Marital Status		
Single	28	59.6
Married/Widowed	19	40.4
Number of Live Births		
None	25	61.0
One or more	16	39.0
Preferred Language of Communication*		
English	27	57.5
Arabic	36	76.6
English & Arabic	16	34.0
Other (Pashto)	1	2.1
Ability to Read and Write in English		
Only Read	1	2.7
Read and Write	33	89.2
Neither	3	8.1
Where do you go when you are sick? *		
Family Doctor	34	72.3
Pharmacy, Clinic, Emergency Room	29	61.7
Nowhere	4	8.5
Where do you go when you need advice about your health? *		
Family Doctor	38	80.9
Pharmacy, Clinic, Hospital	16	34.0
Digital Resources, Family and Friends, Alternative Medicine	29	61.7

Note: Certain demographics do not add up to 100% because participants were able to select all answers that apply. (n=participants)

Most participants preferred to communicate in Arabic, even though most of them could read and write in English. Most participants went to their family physician when they were sick and when they needed health advice.

We found an overall improvement in each literacy item on the survey (Table 2). Compared to the pre-survey, we observed a significant increase in the post-survey on the average score of both eHL [0.266 (t = 2.81, *p* = 0.0073)] and PHL [0.666 (t = 5.62, *p* < 0.0001)]. Nulliparous females under 35 years of age improved in PHL more than those who are 35 years or older, F(1,45) = 4.96, *p* < 0.031; they also started at a lower baseline score for PHL than participants with at least one child. When controlling for age group, nulliparous females showed greater improvement in PHL scores than did parous females (t-2.16, *P*=0.037) (Table 3). The place of birth did not show a noticeable association with the increase in knowledge.

**Table 2:** Comparison of the mean of each literacy item on the pre- and post-surveys

Survey’s Items	Pre-survey Mean	Post-survey Mean
**e-Health Literacy (eHL)**		
eHL1. I know that health resources are available on the Internet.	4.06	4.45
eHL2. I know how to use the Internet to answer my questions about my health.	4.04	4.19
eHL3. I know how to use the health information I find on the Internet to help me.	3.89	4.04
eHL4. I can evaluate the accuracy and credibility of health resources on the internet.	3.62	3.94
eHL5. I can evaluate the accuracy of the health information I hear on TV and radio.	3.49	3.74
eHL6. I feel confident in using information from the Internet to make health decisions.	3.36	3.70
**Average eHL score**	**3.74**	**4.01**
**Postpartum Health Literacy (PHL)**		
PHL1. It is difficult for me to understand the contents of written materials about health and illness.	2.87	2.64
PHL2. I understand what doctors and staff at health centers say about the illness or problem of my sexual health.	3.89	4.06
PHL3. I understand the benefits and side effects of medications or contraceptives recommended by my doctor/healthcare professional.	3.72	4.13
PHL4. I understand when a woman’s body is ready to have children.	3.41	4.33
PHL5. I understand how the reproductive system works.	3.96	4.29
PHL6**.** I know how to get information about healthy nutrition before getting pregnant.	3.43	4.38
PHL7. I can talk to my spouse about how to use family planning (pregnancy prevention).	3.43	4.31
PHL8. I know where to go when I need counseling about married life and dealing with my spouse.	3.03	4.00
PHL9. I know where to go for advice and guidance if I have a sexual problem in my married life.	3.28	3.88
PHL10. I know when and where to go for the necessary tests or examinations when I am pregnant.	3.60	4.48
**Average PHL score**	**3.46**	**4.05**

**Table 3:** Regression results for change in Pre-to-Post PHL scale

Parameter	Estimate	Standard Error	t Value	Pr > |t|	
Intercept	0.07	0.411	0.16	0.874	
Age Under 35	0.12	0.366	0.33	0.741	
Nulliparious	0.83	0.384	2.16	0.037	

r-squared=0.19

Number of observations = 47

We estimated the reliability of the scale with Cronbach’s alpha. The Alpha coefficients for the Raw and Standardized Variables were nearly identical and varied between 0.69 and 0.82 for both the eHL and PHL. The range of the Alpha coefficients in both raw and standardized variables falls under an acceptable-good range (> 0.70), which proves that the scales used were reliable measurements (Raharjanti et al., 2022; Olaniyi, 2019).

## Discussion

This study explored the effectiveness of a group educational session on postpartum literacy in the Arab RIM community. Studies on the Arab immigrant and refugee community in the U.S. are few (Haque, 2015). Because the Arab community is an invisible minority in the U.S., they deserve representation in research as any other community (Awad et al., 2021; Abboud et al., 2019). Arabs are considered an invisible minority as they are usually classified as White while they do not share the same health profile or benefits of White privilege (Abboud et al., 2019). Knowledge of postpartum care is lacking in the general American population, adding to the consequences of immigration and language barriers; the Arab immigrant and refugee population in the country is at a greater disadvantage (Cheng et al., 2006). While most participants reported being able to read and write in English, a considerable portion of participants verbally expressed a preference for discussing the sessions and raising their questions in Arabic. Therefore, despite reported English literacy, language remains a barrier when it comes to comfort and depth of communication. The community’s underrepresentation in research hinges on our ability to understand and provide resources for them, resulting in endless consequences. For example, immigrant Arab females face a higher risk of postpartum depression compared to non-immigrant Arab females, which can negatively affect both mothers’ well-being and child development; group educational sessions on these topics can help by promoting self-care, self-advocacy, and reducing stigma around mental health (Alhasanat & Fry-McComish, 2015; Mughal, 2022). Elements of postpartum care sessions, such as self-care, assertiveness to communicate with the health provider, and identifying accurate information sources, are generalizable to other health topics.

Our study recruitment intended to open education sessions to adults of all ages to promote equal education for the whole community. Usually, education on postpartum care is provided in the context of prenatal care and is limited to females who have just delivered (Albanese et al., 2021). However, our community-based study showed that nulliparous women were also interested in learning about postpartum care. Not surprisingly, nulliparous participants had a lower baseline score than participants who had already delivered an infant. However, nulliparous participants showed higher increases in postpartum literacy.

The results showed that the country of origin was not associated with general and reproductive health literacy. However, Arab RIMs living in the United States may be exposed to many different stressors that may affect their ability to seek healthcare and understand different health topics. Yet, little is known regarding the impact of resident status in Arab Americans on health literacy. This study indicates that using community-catered techniques throughout our group sessions, including pre-recorded presentations and question-and-answer components, is an effective method to improve electronic and reproductive health literacy. Other studies have shown similar results that virtual learning can improve postpartum satisfaction levels in nulliparous females (Mohamadirizi et al., 2013).

According to our survey results, there was a significant increase in knowledge of both eHL and PHL after participation in the group sessions. Whether it was the combination of pre-recorded presentations and the Question and Answer component, the incorporation of bilingual sessions, the use of culturally aware material, or the cultural concordance between the facilitator and participants, our results indicate that the combination of techniques we used improved the knowledge of Arab RIM and is an effective method to do so hereafter.

We encouraged participation across the Arab RIM community by collaborating with different community agencies and mosques during recruitment, intentionally not limiting our cohort to clinical settings. On another note, we achieved an excellent follow-up rate, which we attribute to requesting multiple forms of contact from participants and sending multiple reminders with incentive encouragement to facilitate appropriate post-survey communication. Sharing personal contact information was voluntary, and all but one participant shared at least one form of contact. Participants were initially contacted via text message and email, followed by a phone call if no response was received. Study participants who did not feel comfortable completing the post-survey on their own had the option to receive help from a member of the research team who would administer the survey over the phone and record their responses. Second and third reminders were sent to those who did not respond with reminders about the gift card incentive to further encourage participation. While we do not have data confirming that cultural alignment influenced the high follow-up rate, it remains a strong possibility. Our experience with these methods was met with support from community organizations and has shown that the Arab RIM community can be meaningfully engaged in similar research projects in the future.

The generalizability of the study was limited by the convenience sampling. We reached out to communities across Southern California, which may have resulted in a self-selection bias of participants who are interested in learning about the topic. However, we were able to recruit a diverse group in terms of age, educational level, and country of birth, showing the high potential of engaging the Arab RIM community with an important reproductive health topic. Another limitation is potentially the sample size of 47 participants, which limited the ability to control for potential confounders. As we did not document data about the current pregnancy status, we could not estimate the impact of the current receipt of prenatal care on the effectiveness of the intervention.

The use of pre-recorded presentations in future studies and educational sessions offers a great opportunity to engage the broader community by allowing participants to access resources from home, with little to no cost. Pre-recorded presentations have the potential to offer high-quality and medically accurate sessions in participants’ native language, especially useful in the absence of available bilingual health professionals. This health literacy training approach can be applied to other health topics and other RIM communities (ReproNet, 2024). Therefore, the use of pre-recorded presentations in the sessions we offered can be an effective strategy to increase Arab RIM’s health literacy at the community level, and can be easily replicated with other ethnic groups. Offering health literacy sessions on different topics in weekly intervals allows for reinforcement of health literacy concepts such as the role of self-care, prevention, and identifying and using health information. A study evaluating reproductive health literacy training for Arab and Afghan communities that included three sessions on cervical cancer, family planning, and maternal health/postpartum care found that pre-recorded presentations and didactics with a focus on health literacy, the ability to seek, understand, and use information, improves participants’ general, digital, and reproductive health literacy (Thiel et al., 2025).

To promote postpartum well-being, we piloted a strategy that can be offered to a diverse community of refugees and immigrants in community settings. This intervention could potentially be useful in areas with high numbers of RIMs and thus diverse cultures. Our research results, combined with results from other research studies mentioned above, indicate that the methods used are effective and should be replicated in clinical settings. Continuing efforts to develop similar projects can help encourage open discussion of reproductive health topics, such as postpartum care, within the Arab community.

## Data Availability

The authors confirm that the data supporting the findings of this study are available upon request.

## Material Availability

The authors confirm that the data supporting the findings of this study are available upon request.
